# Supplementary material for: The architecture of functional lateralisation and its relationship to callosal connectivity in the human brain
Source: Nat Commun. 2019 Mar 29;10:1417. doi: 10.1038/s41467-019-09344-1 (PMC6441088; doi:10.1038/s41467-019-09344-1)
Supplement: Supplementary file 6 — Supplementary Information [file 41467_2019_9344_MOESM6_ESM.pdf]

Karolis VR et al. The architecture of functional lateralisation and its relationship to callosal connectivity in the human brain (Supplementary Information)

Supplementary Table 1. Terms selected for the study

acoustic	discriminative	insights	place	shifting
action	disgust	integrate	placebo	shifts
action observation	distance	integrated	placebo controlled	short term
actions	distraction	integrating	planning	signal task
addition	distractor	integration	pleasant	similarities
affective	distractors	integrative	pointing	similarity
ambiguous	distress	intelligence	position	size
anger	dorsal attention	intended	predict	skin
angry	duration	intention	predicted	sleep
anticipated	early visual	intentional	predicting	social
anticipation	eating	intentions	prediction	social cognition
anticipatory	economic	interference	prediction error	social cognitive
anxiety	effort	judgment	predictions	social interaction
appraisal	effortful	judgment task	predictive	social interactions
arithmetic	emotion	judgments	predicts	solving
arm	emotion regulation	language	preference	somatosensory
arousal	emotional	language comprehension	preferences	sound
articulatory	emotional faces	language network	preferential	sounds
association	emotional information	languages	preparation	space
associations	emotional neutral	learn	preparatory	span
associative	emotional responses	learned	primary auditory	spatial
attend	emotional stimuli	learning	primary motor	spatial attention
attended	emotional valence	learning task	primary sensorimotor	spatial information
attending	emotions	letter	primary sensory	spatial temporal

attention	empathic	letters	primary somatosensory	spatiotemporal
attention task	empathy	lexical	primary visual	speaking
attentional	empirical	lexical decision	prime	speech
attentional control	encode	limb	priming	speech perception
attribution	encoded	linguistic	probabilistic	speech production
audio	encoding	listened	probability	speech sounds
audiovisual	encoding retrieval	listening	probe	speed
auditory	endogenous	long term	prospective	spoken
auditory stimuli	episodic	maintain	pseudowords	spontaneous
auditory visual	episodic memory	maintained	punishment	stimulus driven
autobiographical	error	maintaining	reach	stop signal
autobiographical memory	errors	maintenance	reaching	storage
automated	estimation	match	reactivity	strategic
automatic	executive control	matching	read	strategies
autonomic	executive function	matching task	reading	strategy
aversive	executive functions	memories	reappraisal	stress
avoid	expectancy	memory	reasoning	stroop
avoidance	expectation	memory encoding	recall	stroop task
awareness	expectations	memory load	recognition	subtraction
belief	expected	memory performance	recognition memory	success
beliefs	explicit	memory processes	recognition task	successful
believed	exploration	memory retrieval	recognize	suffering
bias	expression	memory task	recognized	suppression

biased	expressions	memory tasks	recognizing	sustained
biases	external	memory wm	recollection	switch
binding	eye	mental imagery	rehearsal	switching
body	eye field	mentalizing	reinforcement	syntactic
calculation	eye fields	mnemonic	relational	tactile
capacity	eye movement	monetary	relevance	tapping
capture	eye movements	monetary reward	remember	target
categories	eyes	money	remembered	target detection
categorization	face	monitor	remembering	taste
category	face recognition	monitored	repeat	term memory
causal	face stimuli	monitoring	repeated	theory mind
choice	faces	mood	repetition	thinking
choices	facial	moral	repetition suppression	thought
choose	facial expression	motion	repetitive	thoughts
cognitive control	facial expressions	motivation	response inhibition	threat
cognitive emotional	familiar	motivational	response selection	threatening
coherence	familiarity	motor	responsiveness	time task
coherent	fear	motor control	retention	timing
color	fearful	motor imagery	retrieval	tom
combination	fearful faces	motor performance	retrieved	tone
combinations	feedback	motor response	reward	tones
combining	feeling	motor responses	reward anticipation	tool
communication	feelings	motor task	rewarding	tools
competing	finger	movement	rewards	touch
competition	finger movements	movements	rhythm	unfamiliar
comprehension	finger tapping	moving	risk	unpleasant
concept	flexibility	multisensory	risky	valence

concepts	flexible	music	rotation	valuable
conceptual	fluency	musical	rule	value
conditioned	food	names	rules	values
conditioning	foot	naming	saccade	verb
conflict	form	navigation	saccades	verbal
conflicting	forms	negative emotional	sad	verbal fluency
congruency	gain	neutral faces	salience	verbal working
congruent	gains	neutral pictures	salient	verbs
congruent incongruent	gambling	neutral stimuli	search	video
conscious	game	nociceptive	secondary somatosensory	video clips
consciousness	gaze	nogo	seeking	videos
consolidation	gestures	noun	segregation	view
context	goal	nouns	selection	viewed
contexts	goal directed	novel	selective	viewing
contextual	goals	novelty	selective attention	violations
control processes	grasping	noxious	selectivity	virtual
coordination	hand	number	self	vision
covert	hand movements	numbers	self referential	visual
craving	hands	numerical	self reported	visual attention
cue	happy	object	semantic	visual auditory
cued	happy faces	object recognition	semantic information	visual field
cues	head	objects	semantic knowledge	visual information
decision	heard	observing	semantic memory	visual motion
decision making	hearing	oddball	semantics	visual perception
decision task	identification	oral	sensation	visual spatial
decisions	identity	order	sensations	visual stimuli

declarative	illusion	orientation	sensorimotor	visual stimulus
decoding	imagery	oriented	sensory	visual word
default mode	imagine	orienting	sensory information	visuo
default network	imagined	orthographic	sensory modalities	visuomotor
delay	imitation	overt	sensory motor	visuospatial
delayed	implicit	pain	sentence	vocal
demand	impulsivity	painful	sentence comprehension	voice
demanding	incongruent	passive viewing	sentences	voluntary
demands	index finger	personal	sequence	wm
depth	induction	personality	sequences	wm task
detect	inference	personality traits	sequential	word
detected	inferences	perspective	serial	word form
detecting	inhibit	phonetic	series	word pairs
detection	inhibiting	phonological	sex	word recognition
detection task	inhibition	photographs	sexual	words
digit	inhibitory	picture	shape	work
discrimination	inhibitory control	pictures	shapes	working memory
discrimination task	insight	pitch	shift	written

Karolis VR et al. The architecture of functional lateralisation and its relationship to callosal connectivity in the human brain (Supplementary Information)

Supplementary Table 2. Taxonomy of functional lateralisation

Maps	Principal Component label	RH > LH				LH > RH			
		# voxels	T value	MNI X, Y, Z	Anatomical area	# voxels	T value	MNI X, Y, Z	Anatomical area
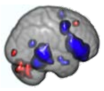	Language	911	18.05	12,-78,-34	Crus II	2947	42.03	-50,18,18	Prefrontal. C., extending into Precentral. G.
		45	12.45	28,28,50	Sup. Front S.	2607	40.59	-58,-42,6	Mid. Temp. G. (posterior) & Supram. G. extending into Fusiform C.
		28	12.41	32,-96,6	Occipital pole	160	20.59	-6,8,62	SMA (medial)
						102	18.03	-40,-60,24	Sup. Temp. S., (posterior, deep)
						98	15.71	-52,-8,-8	Sup. Temp. S. (middle-to-anterior segment)
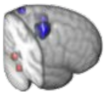	Movement	36	13.30	6,-56,-12	Area V	365	15.53	-40,-14,60	Precentral G. (middle part)
		23	12.24	18,-50,-28	Area VI	149	16.35	-10,-12,58	Border of Precentral G (medial) & SMA
						34	13.78	-10,-44,76	Border of Sup. Par. L. & Postcentral G.
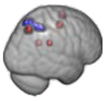	Eye movement	120	16.50	20,-64,52	Sup. Par. L., bank of Intra-Par. S.	322	19.04	-30,-48,56	Sup. Par. L.
		66	15.55	4,8,60	SMA (medial)				
		48	13.15	24,-8,52	Mid. Front. S, (posterior end)				
		25	13.77	24,-46,26	White matter				
		21	11.96	56,-40,38	Supram. G. (posterior)				
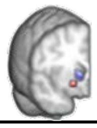	Reward	23	11.41	14,10,-8	Putamen (inferior)	66	20.03	-2,10,-2	Nucleus accumb.*
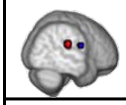	Pain	107	17.53	52,-30,24	Planum temporale	36	13.36	-62,-10,22	Postcentral G. (inferior)
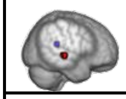	Auditory	60	11.90	58,-20,0	Sup. Temp. G. (posterior)	38	12.44	-48,-34,20	Planum temporale
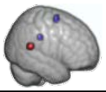	Action (observation)	74	14.48	62,-38,22	Supram. G. (posterior)	59	14.25	-24,0,66	Sup. Front. G., (posterior)
						40	13.33	-56,-22,38	Postcentral S. (inferior) & Supram. G. (anterior)
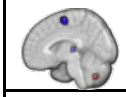	(Finger) tapping	54	13.02	14,-62,-44	Area VIIIb	93	16.36	-4,-6,60	SMA (medial)
						29	13.34	-10,-20,8	Thalamus (posterior)
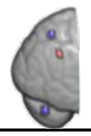	Calculation/numerical	25	13.01	40,-50,42	IPS (lower bank)	59	14.75	-36,-68,-38	Crus I
						35	14.14	-20,-58,56	IPS (upper bank)
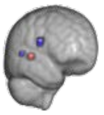	Phonetic/ Speech perception	47	14.01	48,-24,2	Sup. Temp. S. (posterior, deep)	56	12.98	-56,6,18	Precentral G.(inferior)
						20	11.68	-62,-28,4	Sup. Temp. G. (posterior)

Karolis VR et al. The architecture of functional lateralisation and its relationship to callosal connectivity in the human brain (Supplementary Information)

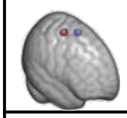	(Motor) imagery	27	12.60	34,-10,72	Precentral G.	21	12.50	-18,4,68	Sup. Front. G.
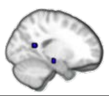	Autobiographical (memory)					26	14.33	-14,-52,16	Precuneus/anterior end of Par.-Occ. S.
						21	11.98	-22,-16,-14	Hippocampus
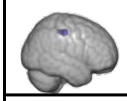	Touch					92	14.32	-34,-28,52	Postcentral G.
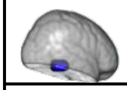	Visual word/form					498	24.20	-42,-42,-12	Fusiform cortex
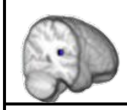	Music					36	12.36	-38,-26,18	Planum temporale
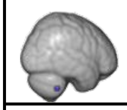	Motor performance					21	12.42	-44,-56,-46	Crus II
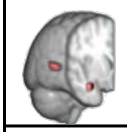	Facial expression	100	14.87	52,-44,4	Sup. Temp. S.(posterior)				
		27	11.96	34,2,-26	Amygdala (inferior)				
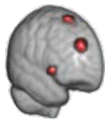	Stop/ inhibition	196	17.84	24,52,34	Anterior segment MFSulc / frontal pole				
		88	13.41	18,16,68	Sup. Front.G.				
		58	13.58	48,22,-2	Pars opercularis (inferior)				
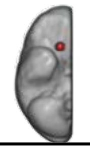	Decision making	44	14.19	14,28,-20	Medial Orbital G. (posterior)				
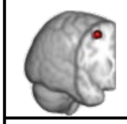	Working memory	54	13.61	32,12,54	Mid. Front G. (posterior)				
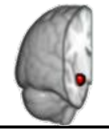	Fearful (faces)	82	17.09	30,0,-14	Amygdala (superior)				
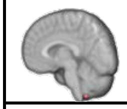	(Un-) pleasant (faces)	29	13.70	8,-50,-60	Area VIIIb				
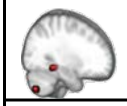	Navigation	50	12.70	36,-66,-50	Area VIIb				
		38	11.99	28,-36,-14	Parahippocampal G. & Fusiform C.				
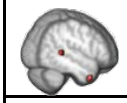	Social interaction	48	12.89	44,10,-38	Temporal pole				
		24	11.90	44,-44,8	Sup. Temp S. (posterior end)				
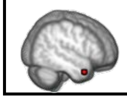	Violations	42	13.16	50,10,-30	Temporal pole				

Supplementary Table 3. Principal components and the terms with highest loadings

Language		Movement		Eye movement	
language	1.24	movement	0.66	eye movements	0.63
semantic	1.24	motor	0.60	eye	0.61
words	1.21	primary motor	0.55	eye fields	0.53
word	1.12	hand	0.50	saccade	0.52
reading	1.11	sensorimotor	0.50	saccades	0.51
sentences	1.05	movements	0.50	eye movement	0.32
sentence	1.04	motor control	0.42	movements	0.27
phonological	1.03	finger	0.40	eye field	0.26
comprehension	1.01	index finger	0.39	gaze	0.21
lexical	0.82	primary sensorimotor	0.39	moving	0.20
**Reward**		**Pain**		**Auditory**	
reward	0.58	pain	0.80	auditory	0.65
monetary	0.56	painful	0.72	auditory visual	0.52
monetary reward	0.48	noxious	0.60	visual auditory	0.43
reward anticipation	0.46	nociceptive	0.46	sounds	0.34
anticipation	0.39	secondary somatosensory	0.38	auditory stimuli	0.31
rewards	0.37	somatosensory	0.35	audiovisual	0.26
motivation	0.29	sensation	0.19	visual	0.21
motivational	0.19	discriminative	0.18	sound	0.20
rewarding	0.19	affective	0.18	speech	0.18

gain	0.16	skin	0.16	phonological	0.18
Action (observation)		(Finger) tapping		Calculation / numerical	
action	0.65	tapping	0.80	calculation	0.53
actions	0.58	finger tapping	0.78	numerical	0.48
action observation	0.56	finger	0.53	arithmetic	0.48
observing	0.31	motor	0.26	numbers	0.32
grasping	0.19	index finger	0.23	digit	0.26
tools	0.19	motor task	0.17	number	0.24
rehearsal	0.18	motor performance	0.15	subtraction	0.22
movements	0.17	sensorimotor	0.15	distance	0.15
tool	0.17	sequential	0.14	solving	0.14
motion	0.16	hand	0.14	size	0.13
Phonetic / speech perception		(Motor) imagery		Autobiographical (memory)	
phonetic	0.54	imagery	0.73	autobiographical	0.93
speech perception	0.50	motor imagery	0.63	autobiographical memory	0.82
speech	0.37	imagined	0.42	memories	0.49
articulatory	0.26	mental imagery	0.39	semantic memory	0.38
speech sounds	0.23	imagine	0.17	personal	0.33
phonological	0.21	motor	0.16	retrieval	0.25
acoustic	0.17	pointing	0.13	remembering	0.24
speech production	0.17	foot	0.13	semantic	0.22
listening	0.16	hands	0.11	self referential	0.22
covert	0.15	perspective	0.11	retrieved	0.21
Touch		Visual word / form		Music	
touch	0.53	visual word	0.73	musical	0.61

tactile	0.51	word form	0.63	music	0.60
somatosensory	0.50	orthographic	0.50	auditory	0.29
primary somatosensory	0.41	word recognition	0.42	listening	0.26
secondary somatosensory	0.26	reading	0.38	pitch	0.20
multisensory	0.21	word	0.25	sounds	0.18
vision	0.18	words	0.24	sound	0.16
finger	0.15	phonological	0.22	acoustic	0.15
sensory	0.15	form	0.21	rhythm	0.15
hand	0.14	letter	0.21	timing	0.13
Motor performance		Facial expression		Stop / inhibition	
motor performance	0.58	expressions	0.65	signal task	0.68
motor task	0.34	facial expressions	0.62	stop signal	0.65
noxious	0.11	facial	0.61	response inhibition	0.60
numbers	0.10	facial expression	0.48	inhibition	0.54
motor	0.10	expression	0.43	inhibitory	0.46
arousal	0.09	emotional	0.28	inhibitory control	0.39
feedback	0.09	happy	0.24	inhibit	0.25
eyes	0.09	face	0.23	nogo	0.19
finger	0.09	faces	0.21	inhibiting	0.13
memory performance	0.09	disgust	0.20	successful	0.12
Decision making		Working memory		Fearful (faces)	
decision making	0.58	memory wm	0.76	fearful	0.61
decision	0.54	wm	0.73	fearful faces	0.56
choices	0.49	wm task	0.58	faces	0.39
choice	0.47	working memory	0.50	neutral faces	0.27

decisions	0.41	memory	0.28	happy	0.23
value	0.32	maintenance	0.26	face	0.23
risky	0.31	numerical	0.13	angry	0.23
choose	0.26	probe	0.13	happy faces	0.21
rewards	0.21	spatial	0.12	emotional	0.20
gains	0.19	maintained	0.11	fear	0.18
(Un-)pleasant (faces)		Navigation		Social interaction	
unpleasant	0.65	navigation	0.76	social interaction	0.66
pleasant	0.65	virtual	0.48	social	0.31
feelings	0.18	spatial	0.21	social interactions	0.20
valence	0.13	executive functions	0.14	social cognition	0.16
affective	0.13	self	0.14	gaze	0.15
emotional	0.12	space	0.11	recall	0.13
sensation	0.11	observing	0.11	language network	0.13
aversive	0.11	binding	0.09	successful	0.13
neutral pictures	0.11	thinking	0.09	illusion	0.12
social cognition	0.10	orientation	0.09	attend	0.12
Violations					
violations	0.48				
moral	0.25				
game	0.22				
spontaneous	0.18				
mental imagery	0.16				
motivation	0.14				
syntactic	0.14				
sensation	0.13				
sensory	0.13				

information		
conflict	0.12	

Karolis VR et al. The architecture of functional lateralisation and its relationship to callosal connectivity in the human brain (Supplementary Information)

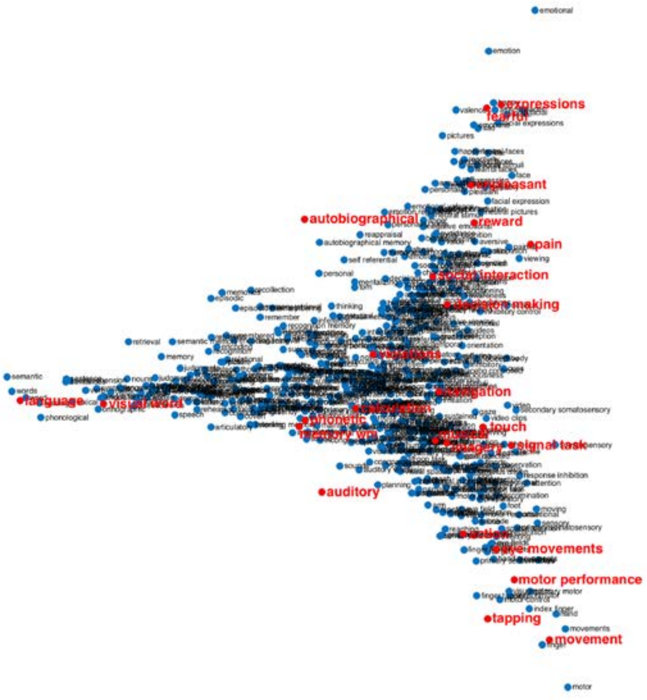

Supplementary Figure 1. Low dimensional structure of the functional lateralisation in the brain. Spatial embedding of all Neurosynth terms in two dimensions revealing a triangular organisation with 3 apices: symbolic communication, perception/action, and emotion.

Karolis VR et al. The architecture of functional lateralisation and its relationship to callosal connectivity in the human brain (Supplementary Information)

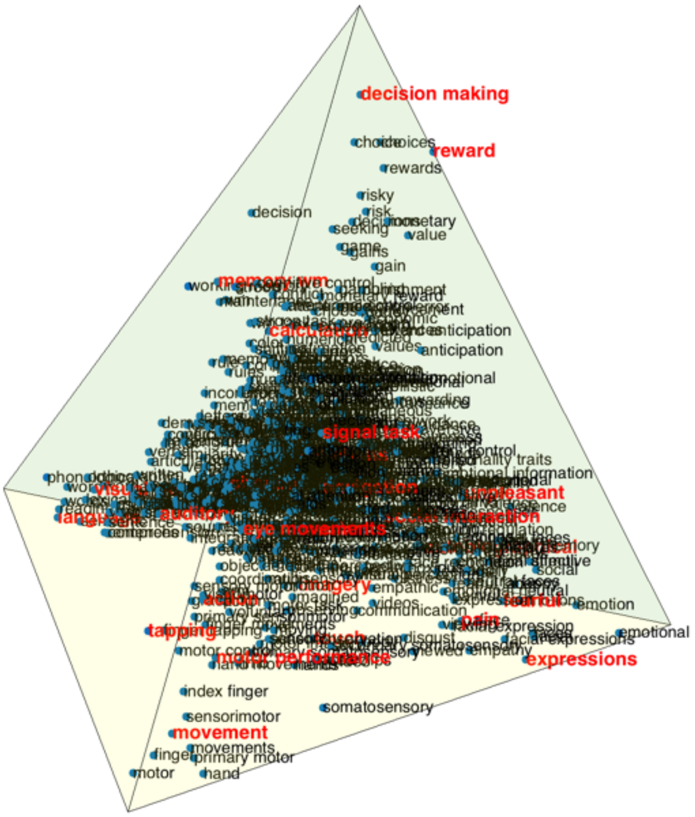

Supplementary Figure 2. Low dimensional structure of the functional lateralisation in the brain. Spatial embedding of all Neurosynth terms in three dimensions revealing a tetrahedron organisation with 4 vertices: symbolic communication, perception/action, emotion and decision making. (see supplementary data 1 for a MATLAB interactive 3D file)

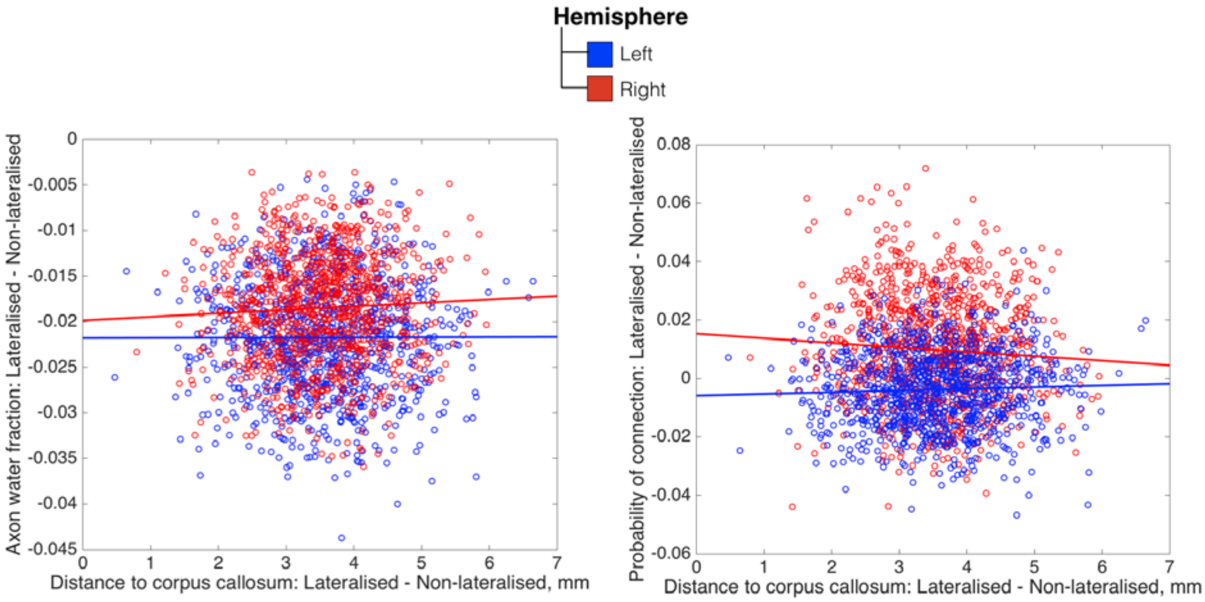

Supplementary Figure 3. Relationship between the two connectivity variables studied in the manuscript and the distance from the midsection of the corpus callosum.

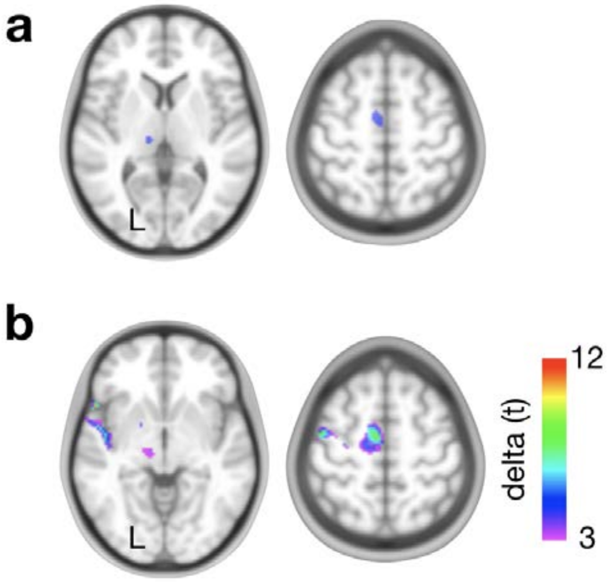

Supplementary figure 4: Validation of the functional asymmetries derived from the meta-analysis of functional MRI with raw functional MRI results derived from a finger tapping task in 142 right-handed participants (Tzouriot-Mazoyer et al. Front Hum Neurosci. 2015). a) functional lateralisation map for finger tapping task derived from the meta-analytic approach b) Subtraction between left-hand and left-right flipped right-hand finger tapping symmetric functional MRI maps.

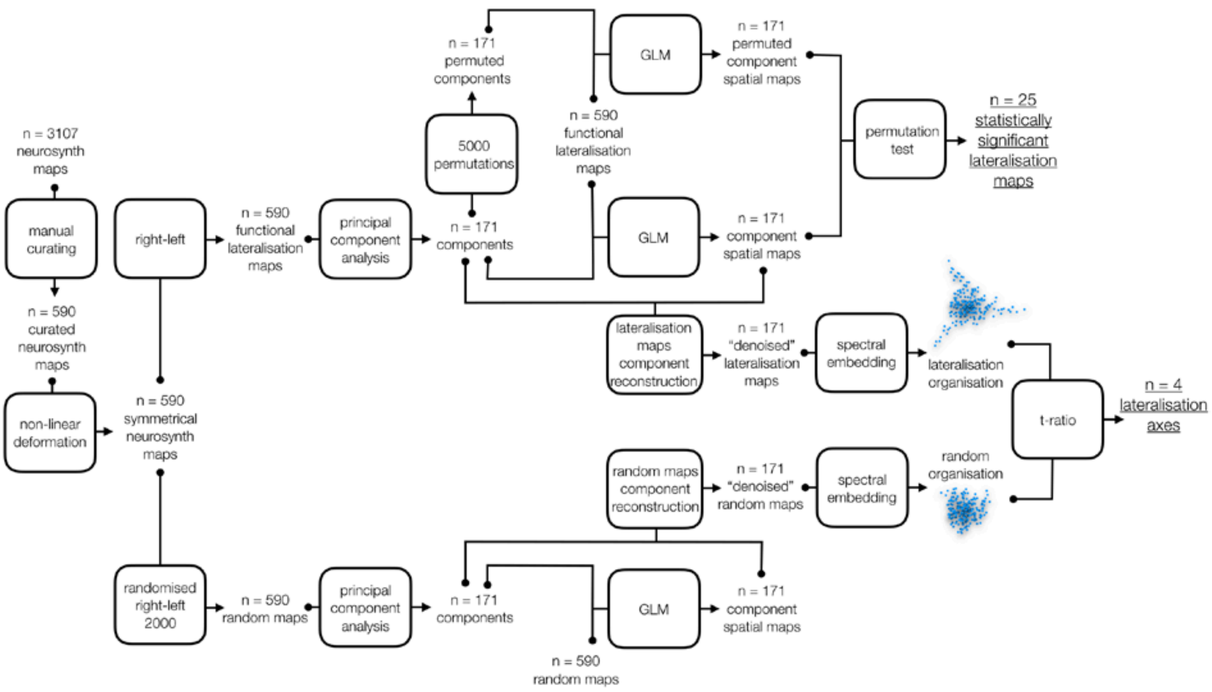

Supplementary Figure 5. Graphical summary of the global structure of functional lateralisation methods

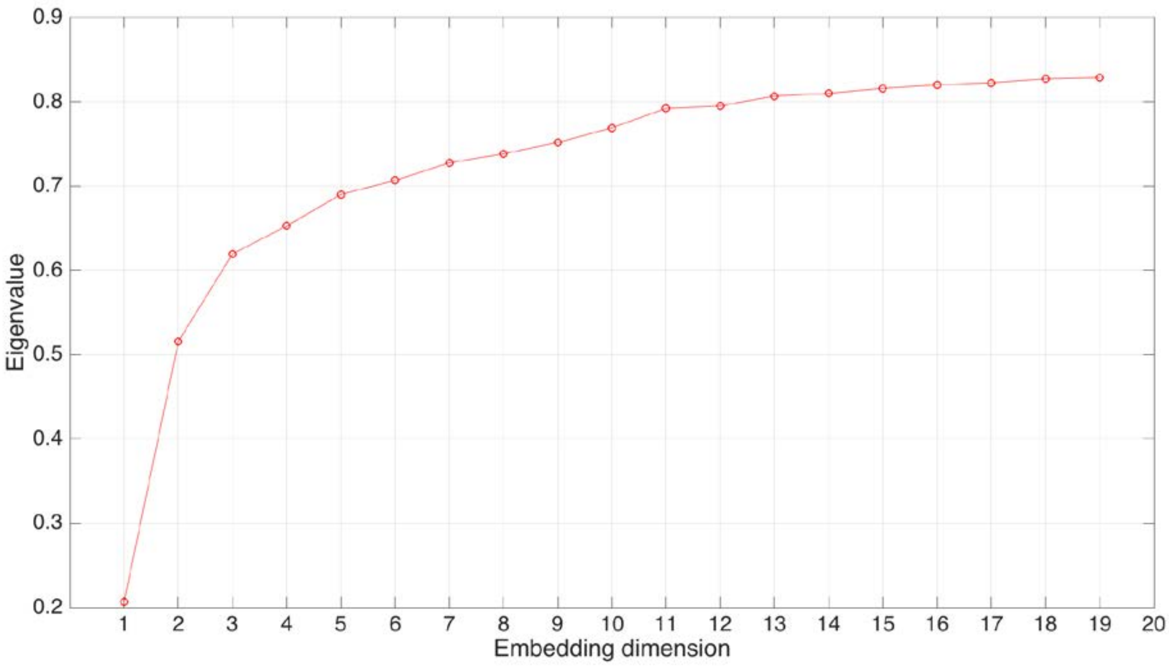

Supplementary Figure 6. Eigenvalues of graph Laplacian embedding

Karolis VR et al. The architecture of functional lateralisation and its relationship to callosal connectivity in the human brain (Supplementary Information)

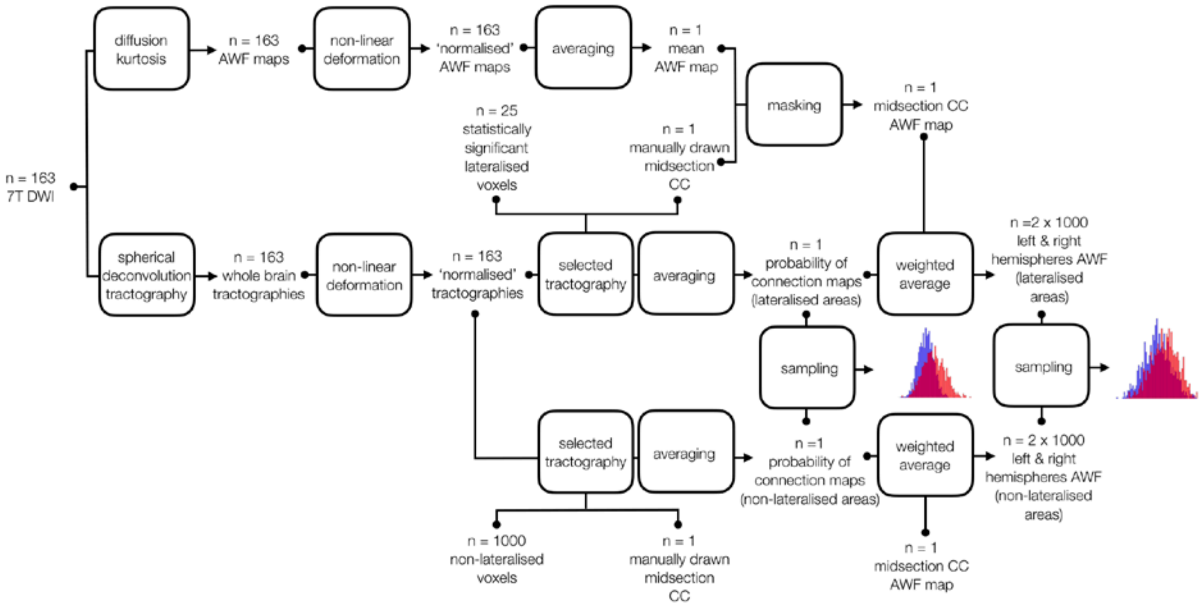

Supplementary Figure 7. Graphical summary of the analyses of function-structure relationship.
