# Supplementary material for: The architecture of functional lateralisation and its relationship to callosal connectivity in the human brain
Source: Nat Commun. 2019 Mar 29;10:1417. doi: 10.1038/s41467-019-09344-1 (PMC6441088; doi:10.1038/s41467-019-09344-1)
Supplement: Supplementary file 7 — Description of Additional Supplementary Files [file 41467_2019_9344_MOESM7_ESM.docx]

Description of Additional Supplementary Files

**Supplementary Data 1:** MATLAB interactive 3D file. Spatial embedding of all Neurosynth terms in three dimensions revealing a tetrahedron organisation with 4 vertices: symbolic communication, perception/action, emotion and decision making.

**Supplementary Data 2:** Archetype maps corresponding to the symbolic communication axis.

**Supplementary Data 3:** Archetype maps corresponding to the perception/action axis.

**Supplementary Data 4:** Archetype maps corresponding to the emotion axis.

**Supplementary Data 5:** Archetype maps corresponding to the decision axis.
